# Can maternal urinary and serum carbohydrate antigen 19-9 concentrations be utilized in the diagnosis of fetal hydronephrosis?

**DOI:** 10.4274/jtgga.galenos.2019.2019.0101

**Published:** 2020-03-06

**Authors:** Murat Akbaş, Faik Mümtaz Koyuncu, Burcu Artunç Ülkümen, Fatma Taneli, Habib Özdemir

**Affiliations:** 1 Department of Obstetrics and Gynecology, Division of Perinatology, Manisa Celal Bayar University Faculty of Medicine, Manisa, Turkey; 2 Department of Medical Biochemistry, Manisa Celal Bayar University, Manisa, Turkey

**Keywords:** Hydronephrosis, pregnancy, CA 19-9

## Abstract

**Objective:**

Fetal hydronephrosis (FH) is the most common fetal renal pathology encountered in daily obstetric practice. Urinary and serum carbohydrate antigen 19-9 (CA 19-9) concentrations are elevated in obstructive renal pathologies. Our aim was to assess maternal urinary and serum CA 19-9 concentrations in pregnancies with FH and compare results with controls.

**Material and Methods:**

Twenty pregnancies with severe FH, 20 pregnancies with mild-moderate FH, and 20 healthy singleton pregnancies were included in this descriptive, case-control study. The diagnosis and classification of FH was based on the anterioposterior diameter of fetal renal pelvis. Maternal urinary and serum CA 19-9 concentrations were measured and compared between groups.

**Results:**

Severe FH cases had significantly higher maternal urinary CA 19-9 concentrations compared to controls (median: 75 vs 24 U/mL; respectively; p=0.014). Concentrations of CA 19-9 did not differ between the mild-moderate FH group and control group. No statistically significant difference was found between the groups with respect to maternal serum CA 19-9 concentrations.

**Conclusion:**

Our results show that maternal urinary CA 19-9 concentration is significantly higher in pregnancies with severe FH. However, no difference was detected in serum CA 19-9 concentrations between pregnancies with severe FH, mild-moderate FH and controls. If the mechanisms of transplacental passage and maternal urinary excretion are clarified, maternal urinary CA 19-9 may be a potential marker for indicating fetal kidney damage.

## Introduction

Fetal renal pathologies are frequently encountered in obstetric practice. Fetal hydronephrosis (FH) is the most common antenatally detected renal pathology (1). The severity of hydronephrosis and the need for postnatal treatment are closely associated with the underlying pathology. The most basic and frequently used technique for evaluation of the fetal renal pelvis is the imaging-based measurement from anterior to posterior in the transverse plane. This measurement depends on the operator and is affected by maternal hydration. Furthermore, it does not provide information about renal parenchymal damage (2,3). Given all these factors, the anterio-posterior measurement of the renal pelvis has low prognostic value. Other ultrasonographic measures and urinary markers are not sufficient to make an accurate diagnosis and predictive enough for clinically significant FH (4).

Carbohydrate antigen 19-9 (CA 19-9) is a Lewis blood group antigen derivative glycoprotein (5). This antigen is found in the amniotic fluid in high concentrations due to the secretion of amnion and decidual cells (6). In addition to being a tumor marker used in gastrointestinal cancers, this antigen is increased in many benign conditions. Renal epithelium excretes CA 19-9 under physiological conditions. High serum and urine concentrations can be detected in patients with severe hydronephrosis (7,8,9). It has been shown that urinary and serum CA 19-9 can be used as a marker for the diagnosis and management of obstructive renal pathologies (10,11).

This study was based on the hypothesis that increased levels of CA 19-9 could be detected in maternal serum and urine due to increased excretion by the fetus with hydronephrosis. The research aim was, therefore, to evaluate CA 19-9 concentrations in maternal serum and urine in pregnancies with FH.

## Material and Methods

Sixty pregnant women who were admitted to the Department of Obstetrics and Gynecology, Manisa Celal Bayar University, between July 2017 and July 2018 were enrolled for this case-control study. Three groups were defined as severe FH, mild-moderate FH and control with each group consisting of twenty cases.

For the diagnosis of mild-moderate hydronephrosis, the criteria of anterioposterior diameter between 4 and 10 mm (second trimester) or 7 and 15 mm (third trimester) were used. Greater than 10 mm and 15 mm were used for the diagnosis of severe hydronephrosis (second and third trimester, respectively) (12). The control group consisted of twenty healthy pregnant women matched for age and gestational age. Obstetric ultrasonography and fetal renal pelvis measurements were performed by a single ultrasonographer with 13 years of experience on grayscale ultrasonography (M.A.). The Health Ethics Board of Manisa Celal Bayar University approved this study (approval number: 20.478.486-23248, date: 28.06.2017). Informed consent was given by all participants and the study was carried out in compliance with the Declaration of Helsinki.

Exclusion criteria were: multiple gestation; any fetal chromosomal or structural anomaly; pregnancies complicated with any type of diabetes mellitus; gestational or pregestational hypertensive disorders; women with inherited thrombophilia, connective tissue disorders, chronic renal or hepatic disease; and history of any proven or suspected malignancy.

A complete obstetric history and demographic data were obtained. Antenatal detailed anomaly scan was performed. Maternal peripheral venous blood and urine samples were collected before 10.00 am following an overnight fast. Store samples tightly stoppered at room temperature and were examined within eight hours. Venous blood samples were centrifuged for 15 minutes at 3000 g. Quantitative measurement of urinary and serum CA 19-9 concentrations was performed using original reagents by a two-region immunoenzymatic immunoassay on the Beckman Coulter Unicel DXI 800 analyzer, [Beckman Coulter, Brea, CA, United States of America (USA)]. The lowest detectable level of CA 19-9 distinguishable from zero with 95% confidence is 0.8 U/mL. This assay exhibits total imprecision of less than 10% across the assay range. One study, using commercially available human serum based control material generating a total of 20 assays, 2 replicates per assay, over 20 days provided 6.4% coefficient of variation for intra-assay precision and 5.7% coefficient of variation for inter-assay precision (13).

### Statistical analysis

Statistical Package for the Social Sciences program, version 20.0 (SPSS Inc., Chicago, IL, USA) was used for statistical analyses. Distribution of variables was assessed with the Shapiro-Wilk test. Statistical comparisons were performed with the ANOVA test (normally distributed data) and the Kruskal-Wallis test or the Mann-Whitney U test (skewed data). Appropriate post-hoc test was utilized for multiple comparisons between groups (Bonferroni and Dunn’s). Chi-square test was utilized for categorical variables. The relationship between the maternal urinary and serum CA 19-9 levels and other parameters were determined with Spearman’s rho correlation coefficient. Normally distributed data were reported as mean ± standard deviation, whereas skewed data were presented as median and range. All reported p values are two-tailed. Statistical significance was assumed when the p value was <0.05.

## Results

**Figure 1:**
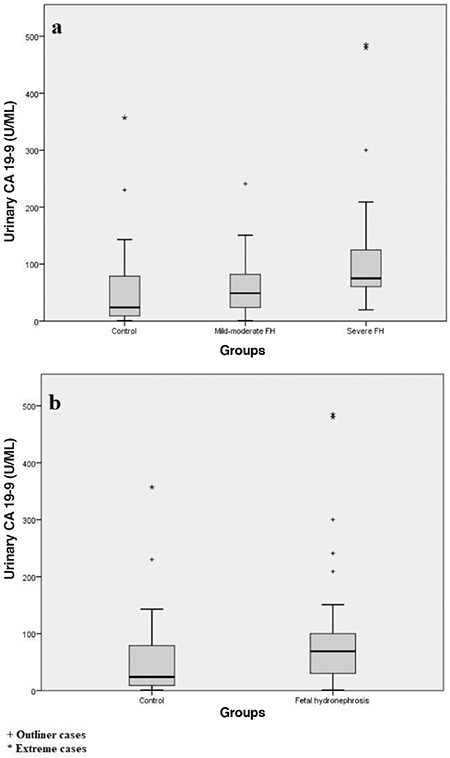
a. Urinary CA 19-9 levels between three groups, b. Urinary CA 19-9 levels between all FH group and controls. Distributions are compared with box plots graphs. FH: Fetal hydronephrosis, CA: Carbohydrate antigen

Maternal age, gestational age and fetal gender were not significantly different between groups (Table 1). Male fetuses constituted the majority in all groups (70%, 65%, 65%). Unilateral renal involvement was more frequent in both hydronephrosis groups (80% vs 65%). No statistically significant difference was found for maternal serum CA 19-9 concentrations between the groups (p=0.353). However, maternal urine CA 19-9 concentrations were elevated in both FH groups (Table 1 and Figure 1). Post-hoc Dunn’s analysis revealed urinary CA 19-9 concentrations were statistically significantly higher in the severe FH group compared to the control group (median: 75 vs 24 U/mL respectively; U=8.6, p=0.014). There was no significant difference between the mild-moderate and severe FH groups (p=0.189). Also, the difference between the mild-moderate FH group and controls did not reach statistically significant level.

To compare the possible diagnostic utility of urinary CA 19-9 for FH, we combined the two FH groups (n=40) and compared with the control group (n=20) in terms of CA 19-9 levels in urine. The Mann-Whitney U test indicated that urinary CA 19-9 concentrations were significantly higher in all women carrying fetuses with hydronephrosis than for women with normal pregnancies (mean rank: 32.53 vs 21.95, respectively; U=227, p=0.023) (Figure 1).

As seen in Table 2, the serum and urinary CA 19-9 concentrations were positively and significantly correlated (r=0.433, p=0.001). In addition, maternal serum and urinary CA 19-9 concentrations were not found to be associated with maternal age, gestational age, gravidity or fetal gender.

## Discussion

Fetal renal obstruction may be transient or permanent and partial or complete. If the pathological process is persistent in nature, nephrogenic tissue may be affected resulting in cystic dysplasia (14). Therefore, differentiation of renal pathology is important in determining the outcome. Intuitively, FH may be considered as an obstructive pathology. However, in some cases, FH can be the result of non-obstructive processes, such as vesicoureteral reflux or megaureter (15). However, the differentiation may not be possible until delivery. Current diagnostic methods are not adequately predictive to differentiate postnatal clinically significant cases of FH, and in this circumstance, the antenatal management of this condition is challenging and controversial.

Different serum and urine markers have been described for monitoring hydronephrosis and its treatment in children, and utilization of such markers during the antenatal period may be promising in the detection of fetuses requiring treatment (16). CA 19-9 is an antigen of oncofetal origin, produced by amnion cells and decidua. Therefore, the concentration of this antigen in amniotic fluid is high and increases as the gestational period progress (6). Oncofetal antigens may pass from the embryoplacental compartment into the maternal circulation (17). The amount of antigen in maternal circulation depends on factors such as renal function, half-life, molecular weight and protein characteristics. The maternal serum concentration of CA 19-9 is increased throughout pregnancy but does not exceed normal values (0-35.3 U/mL), hence CA 19-9 could be a useful biomarker during pregnancy (18). Maternal serum CA 19-9 levels were found to be significantly higher in primigravidity, female fetus and fetal aneuploidy (19,20). In the current study, groups were homogenous and not significantly different in terms of gestational age, gravidity and fetal gender.

Inflammation and persistent obstruction in the kidney can lead to increased concentrations of CA 19-9 in circulation and in urine (21,22). In line with studies indicating increased excretion of CA 19-9 in obstructive renal pathologies, it can be hypothesized that CA 19-9 concentration in amniotic fluid may be higher in cases of FH (10,11). Newborns diagnosed with a posterior urethral valve have been found to show elevated concentrations of CA 19-9 in urine samples taken immediately after birth and high levels were reported to be associated with a poor prognosis (23). In addition, higher concentrations of amniotic fluid CA 19-9 were reported in pregnancies with posterior urethral valves (24). In the light of this information, we aimed to evaluate maternal serum and urine CA 19-9 concentrations in pregnancies with FH.

In our study, there was a statistically significant difference between severe FH and the control group in terms of urinary CA 19-9 concentration. Our finding was consistent with Kajbafzadeh et al. (25) who reported that CA 19-9 excretion increased significantly in the urine of pregnant women with severe FH. In the current study, the pairwise analysis also revealed an insignificant difference between mild-moderate FH and controls. We did not define a cut-off point for the diagnosis of severe FH because of the small number of cases and skewed distribution. Unspecified maternal renal disease or physiological renal changes due to pregnancy may be the factors affecting urinary excretion of CA 19-9 that led to outlier results and skewed distribution. When we compared the urinary CA 19-9 concentrations between controls and all FH cases (n=40), the difference was still statistically significant. However, the skewed distribution of the data, the very wide ranges of measurements of CA 19-9 even in the control group and insignificant difference between mild-moderate FH and controls make this marker useless to discriminate all FH cases.

It was interesting that there was no statistically significant difference between groups for maternal serum CA 19-9 levels. Transplacental passage of CA 19-9 into maternal circulation has been described in previous studies (19,20,26). Our result may be due to the fact that, even though an increased concentration of the antigen may have been present in amniotic fluid it might be eliminated quickly in urine after transplacental passage. Measurement of amniotic fluid CA 19-9 and comparing the results with maternal serum and urine CA 19-9 levels might clarify this fact. Transplacental passage and ultimate maternal urinary excretion of CA 19-9 is intuitive, since, to date, it has not been studied by either in- or ex-vivo studies. Also, the difference in serum concentrations may fail to reach a statistically significant level due to the small sample size in our study. Last but not least, it should be kept in mind that CA 19-9 is not expressed in 7% of people (27). But, elevated levels of CA 19-9 could be found in some pathological conditions regardless of the Lewis phenotype (28). Even so, the unknown Lewis phenotype of mothers nor fetuses would limit the utility of the marker.

The strength of our study was the homogeneity of the groups with regards to maternal age, gravidity, fetal gender and gestational week at sampling. However, our investigation has several limitations. A key limitation was the small number of cases in each group. Although mild FH is not a rare antenatal finding, severe and progressive cases are. In the unilateral FH cases, the diluting function of normal kidney can affect CA 19-9 measurement but due to the small sample size, we could not sub-divide groups further. Another limitation of the study was that amniotic fluid levels of CA 19-9 were not evaluated. Furthermore, cases with progressive hydronephrosis requiring treatment were not identified because of a lack of neonatal outcomes. One future research objective would be to assess the correlation between increased maternal urinary CA 19-9 and neonatal urinary CA 19-9 concentrations.

## Conclusion

Our findings suggest that this non-invasive diagnostic method could detect clinically significant severe FH but the descriptive nature of the study prevents us from identifying a clinical significant cut-off concentration for maternal urinary CA 19-9 concentration in FH. Also, there may be many subtle maternal variables affecting urinary excretion of CA 19-9 which may result in high concentrations in maternal urine in the absence of FH, thus interfering with the diagnostic accuracy. It might be more accurate to improve the ultrasonographic assessment techniques). Therefore, with the available data, we can not make precise comments about the use of CA 19-9 as a reliable diagnostic marker for FH. Further longitudinal and comprehensive studies with much larger sample sizes are needed to identify the clinical significance of maternal urinary CA 19-9 level in cases of FH.

## Figures and Tables

**Table 1:**
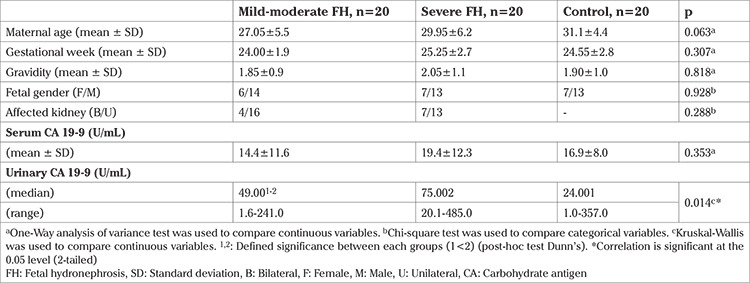
Maternal clinical and biochemical characteristics of study groups

**Table 2:**
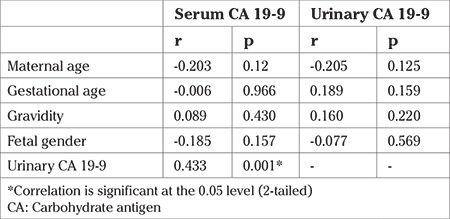
Correlations between serum and urinary carbohydrate antigen 19-9 levels and the other parameters
